# P086 When steroids mislead: an unexpected cause of steroid-responsive temporal headache

**DOI:** 10.1093/rap/rkag113.116

**Published:** 2026-11-03

**Authors:** Elsa Sunny, Nirooshika Illanko, Alexandra Godlee, Elizabeth Rankin

**Affiliations:** Queen Elizabeth Hospital, Birmingham, United Kingdom; Queen Elizabeth Hospital, Birmingham, United Kingdom; Queen Elizabeth Hospital, Birmingham, United Kingdom; Queen Elizabeth Hospital, Birmingham, United Kingdom

## Abstract

**Introduction:**

Giant cell arteritis (GCA) is a critical diagnosis to exclude in older adults presenting with new-onset headache and jaw claudication, given the risk of irreversible visual loss. Although prompt corticosteroid therapy is standard practice, a favourable clinical response should not be regarded as diagnostic. Extranodal diffuse large B-cell lymphoma (DLBCL) involving the craniofacial soft tissues is uncommon and may closely mimic GCA. We describe an elderly patient with stage IV DLBCL whose presentation strongly suggested GCA but was ultimately attributed to temporal lymphoma deposit. This case highlights the importance of maintaining diagnostic vigilance when clinical or investigative findings are atypical.

**Case description:**

A man in his eighties with stage IV diffuse large B-cell lymphoma (DLBCL), previously treated with R-CVP chemotherapy and radiotherapy to the right axilla, was referred with suspected giant cell arteritis (GCA). Two weeks before presentation, his wife had noticed mild swelling over the right temple and cheek, which was neither painful nor concerning to the patient. He subsequently developed a new right-sided temporal headache, followed by jaw pain on mastication, raising suspicion for GCA. He denied visual disturbances, diplopia and scalp ulceration.

In primary care, prednisolone 40 mg daily was commenced resulting in rapid symptomatic improvement. Given the modest inflammatory response (CRP 13mg/L), corticosteroids were discontinued after five days. Within 24 hours of stopping treatment, his headache recurred with increasing severity, prompting urgent Rheumatology assessment.

Examination demonstrated persistent right temporal and zygomatic soft tissue swelling with focal tenderness. Temporal artery ultrasound was negative for features of vasculitis. Re-evaluation of a surveillance FDG PET-CT performed several weeks earlier revealed a focal hypermetabolic lesion adjacent to the right orbit, corresponding to the site of maximal symptoms. Contrast-enhanced MRI confirmed extranodal lymphoma involving the right temporal region with extension into the soft tissues overlying the anterior aspect of the right zygoma. Corticosteroids were dropped to a lower dose, and subsequently tapered. Management was redirected towards relapsed lymphoma.

**Discussion:**

This case illustrates the diagnostic complexity of evaluating new cranial symptoms in an older patient with a background of haematological malignancy. The initial presentation of temporal headache and jaw claudication, coupled with a prompt response to corticosteroids, was highly suggestive of GCA. However, mild local swelling, relatively low inflammatory burden and absence of supportive vascular imaging findings prompted further diagnostic review.

The case highlights an important clinical principle: improvement with corticosteroids is not specific to GCA and should not be considered confirmatory. Lymphoma deposits can demonstrate significant steroid responsiveness due to their lymphoid origin and may therefore mimic inflammatory rheumatological conditions. In this patient, careful correlation of clinical findings with prior FDG PET-CT imaging was pivotal in identifying an alternative diagnosis and avoiding prolonged immunosuppression without addressing the underlying pathology.

For rheumatologists, this case reinforces the importance of maintaining diagnostic flexibility when assessing suspected GCA, particularly in patients with known malignancy or atypical features. A structured approach incorporating clinical phenotype, inflammatory markers, vascular imaging and review of existing oncological investigations can help distinguish vasculitis from important mimics.

**Key learning points:**

1. Corticosteroid responsiveness is not diagnostic of giant cell arteritis and should not replace careful clinical reassessment when features are atypical.

2. In patients with a history of malignancy presenting with new cranial symptoms, lymphoma infiltration should be considered as an important mimic of inflammatory vasculitis.

3. A multidisciplinary approach, integrating rheumatological assessment with review of oncological imaging, can be crucial in identifying alternative diagnoses and avoiding unnecessary prolonged immunosuppression.

